# Blood Pressure Estimation from Photoplethysmogram Using a Spectro-Temporal Deep Neural Network

**DOI:** 10.3390/s19153420

**Published:** 2019-08-04

**Authors:** Gašper Slapničar, Nejc Mlakar, Mitja Luštrek

**Affiliations:** 1 Jožef Stefan Institute, 1000 Ljubljana, Slovenia; 2 Faculty of Computer and Information Science, University of Ljubljana, 1000 Ljubljana, Slovenia; 3 Jožef Stefan International Postgraduate School, 1000 Ljubljana, Slovenia

**Keywords:** blood pressure, photoplethysmogram, deep learning, signal processing, regression

## Abstract

Blood pressure (BP) is a direct indicator of hypertension, a dangerous and potentially deadly condition. Regular monitoring of BP is thus important, but many people have aversion towards cuff-based devices, and their limitation is that they can only be used at rest. Using just a photoplethysmogram (PPG) to estimate BP is a potential solution investigated in our study. We analyzed the MIMIC III database for high-quality PPG and arterial BP waveforms, resulting in over 700 h of signals after preprocessing, belonging to 510 subjects. We then used the PPG alongside its first and second derivative as inputs into a novel spectro-temporal deep neural network with residual connections. We have shown in a leave-one-subject-out experiment that the network is able to model the dependency between PPG and BP, achieving mean absolute errors of 9.43 for systolic and 6.88 for diastolic BP. Additionally we have shown that personalization of models is important and substantially improves the results, while deriving a good general predictive model is difficult. We have made crucial parts of our study, especially the list of used subjects and our neural network code, publicly available, in an effort to provide a solid baseline and simplify potential comparison between future studies on an explicit MIMIC III subset.

## 1. Introduction

Blood pressure (BP) measurement is the most important commonly performed medical office test [1]. BP is a direct indicator of hypertension, an important risk factor for a variety of cardiovascular diseases (CVDs), which were the most common cause of death in 2015, responsible for almost 15 million deaths worldwide according to the World Health Organization (WHO) [2]. Regular BP monitoring is thus important for the general population, but especially vital for people already suffering from hypertension or related conditions, as such people are particularly vulnerable to elevated BP [3].

Despite its importance, an obvious aversion towards regular BP monitoring is present. This can in large part be attributed to the nature of the measuring devices. Cuff-based devices remain the golden standard and are commonly recommended by physicians [3]. These devices offer the highest measurement accuracy, however, they also have several downsides. The person using a cuff-based device must follow a relatively strict measuring protocol in order to ensure the measured values are correct [4]. The measuring procedure can be tedious and requires dedicated time and effort. Physical activity (e.g., exercise) typically does not allow for simultaneous measuring of BP with a cuff. Furthermore, the measuring event itself can cause stress or anxiety in the subject, which can in turn influence the measured BP values. This is commonly known as the white-coat syndrome [5].

Due to the aforementioned factors, work in this domain focuses on developing robust unobtrusive BP estimation systems, which can offer near real-time periodic BP updates to the user. The catalyst for the development of such systems is the increasing presence of wearable devices such as wristbands, which can collect a myriad of physiological signals in a rather unobtrusive way [6]. One such signal, which characterizes the state of the cardiovascular system, is the photoplethysmogram (PPG), which we describe in more detail in the following section.

### 1.1. PPG Background

Photoplethysmography is a rather simple and inexpensive technology, which finds its prevalent medical use in measuring the heart rate and oxygen saturation [7]. It is based on illumination of the skin and measurement of changes in its light absorption. It is typically implemented with a light-emitting diode (LED) to illuminate the skin and a photodetector (photodiode) to measure the amount of light either transmitted through, or reflected from the skin [8].

**Figure 1:**
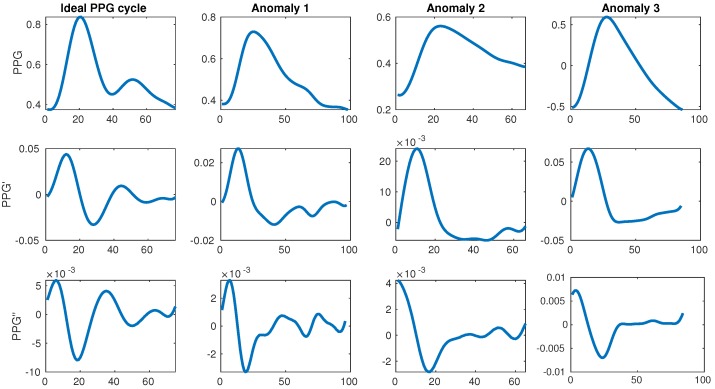
Ideal photoplethysmogram (PPG) cycle waveform and its first and second derivatives next to distorted waveforms. The ideal example has a single large systolic peak and a single lower diastolic peak afterwards, while the anomalies have too many or too few peaks. All data is taken from the MIMIC III database [11].

The change in color and consequently light absorption of the tissue is governed by the blood circulation in the body, which is driven by the systole and diastole of the beating heart. A person’s BP increases as a consequence of the heart muscle contracting during systole, which pushes the blood towards the periphery of the body. This blood pulse wave propagation results in an increased BP. Similarly, the BP decreases when the heart relaxes to fill with blood during the diastole. This results in a periodic signal with clear and explicit systolic peaks (first traversal of blood as it is pushed from the heart during the systole) and subtle diastolic peaks (blood returning from the periphery towards the heart during the diastole). Each periodically-repeated cycle contains a single systolic and a single diastolic peak. The former is typically easily detected and can serve to determine the heart rate (HR) while the latter is sometimes explicit, but more often it can be very difficult to properly detect. Examples of an ideal waveform and common anomalies are shown in Figure 1.

The relationship between the PPG and BP is well established if the PPG is used to measure the speed of the blood flow, commonly known as pulse wave velocity (PWV) [9]. When vessels are stiffer or more contracted, the blood travels faster and exerts more pressure. Similarly, when vessels are more relaxed or elastic, the blood travels more slowly and exerts less pressure. The time it takes for a blood pulse originating from the heart to reach a peripheral point of the body is called pulse transit time (PTT) [10]. When the PTT is shorter, this indicates a higher BP, while a longer PTT indicates a lower BP. However, in order to use this approach, two sensors are needed, typically an electrocardiogram (ECG) at the heart and a PPG at a peripheral point of the body.

Subtle variation in blood volume during heart activity, which is reflected in the morphology of the PPG signal, also appears to be related to BP. However, this relation is currently only modelled with machine learning. Deriving such a model is subject of intense recent research efforts discussed in Section 1.3, as well as our work.

### 1.2. PTT Approach to BP Estimation

The mathematical foundation and correlation between the PWV and vascular stiffness was first studied and formalized by Bramwell et al. in 1922 [9]. Two copper pipes were connected to an artery and mercury was pushed in waves through the device. Mercury was chosen instead of actual blood due to the fact that the artery segment was very short and subsequently the time-interval available for the measurement of the PWV was also very short.

The specific connection between arterial BP (ABP) and PTT was initially analyzed by Geddes et al. in 1981 [10]. They evaluated the relationship between pulse arrival times and diastolic BP (DBP) in 10 anesthetized dogs. The dogs’ BP was chemically manipulated and the time it took for the R peak of the ECG signal, corresponding to the systole, to show in carotid and femoral pulses was measured. A good near-linear correlation was found between changes in PTT and DBP.

Chan et al. created a simple system for cuffless BP estimation system to be used in telemedicine in 2001 [12]. An unspecified number of subjects participated in a laboratory experiment, where their ECG and PPG were measured at a sampling rate of 1500 Hz, and their ground-truth BP was calculated with a cuff-based device. PTT was measured, and 20 sessions per subject were used for calibration. Following this calibration, a linear relationship between PTT and BP was shown. They used the mean error (ME) in milimeters of mercury (mmHg) between the actual and predicted BP as the evaluation metric, and achieved a ME of 7.5 for systolic BP (SBP) and 4.1 for DBP. The usage of ME for evaluation can be problematic, as similarly large positive and negative differences cancel each other out in the overall mean, showing a low overall ME even if individual MEs are large.

One of the recent examples of the PTT approach, which is similar in scope to our own work, was published by Kachuee et al. in 2017 [13]. BP was estimated from roughly a thousand subjects from the MIMIC II database. Their pre-processed PPG and ECG were used to derive PTT-related features and ABP was used as the ground truth. Several regression models were evaluated in a 10-fold cross validation (CV) experiment. The best results were achieved using Adaptive Boosting (AdaBoost), specifically the mean absolute error (MAE) of 11.17 for SBP and 5.35 for DBP. They also used person-specific calibration in their work, which improved the results. The reported evaluation metrics are more suitable compared to the previously mentioned work and give a better insight into the results.

The recent rise of deep learning, which is shown to work extremely well on a myriad of domains, is also reflected in this research area. Su et al. published a paper in 2018 [14] where they highlighted the problem of accuracy decay of existing models for BP estimation from PPG over long time periods. This problem means that frequent calibration is required. A deep recurrent neural network (RNN) with long short-term memory (LSTM) was used to model temporal dependencies in BP. PPG and ECG were used as inputs, and PTT alongside some other features was used to predict BP. They showed long-term improvements in BP prediction accuracy compared to existing methods, while keeping real-time prediction accuracy at a level comparable to related work.

The PTT approach was thoroughly tested in the past 10 years [15,16] and a number of slight variations were proposed, however, the underlying principle remains unchanged. The downside of this method is the fact that two sensors are required to measure the PTT, making it less convenient than single-device approaches. This also means that two signals must be monitored, pre-processed and analyzed. Finally, since the same pulse wave must be traced, precise synchronization and peak detection between the two (often wireless) sensors are vital, requiring a lot of effort before the processing may even commence.

### 1.3. PPG-Only Approach to BP Estimation

The downsides mentioned in the previous section, alongside the increasing presence of wearable devices, have given rise to new research using a single PPG signal. This research focuses on the analysis of the PPG morphology, with the help of features that commonly describe the shape of the PPG waveform on a per-cycle basis.

One of the earliest attempts at BP estimation using only the PPG signal was conducted by Teng et al. in 2003 [17]. They analyzed the relationship between ABP and certain features derived from the PPG waveform. The data was collected from 15 young healthy subject using professional equipment in a highly controlled environment, ensuring constant temperature, no movement and silence. Feature selection was conducted using correlation analysis, and a linear regression algorithm was used to model the relationship. The ME between the estimated and ground truth BP values was 0.21 ± 7.32 mmHg for SBP and 0.02 ± 4.39 mmHg for DBP. Using ME instead of MAE is once again a potential issue, due to reasons described before. Additionally, the sample size was small and the conditions were highly controlled, so it is questionable how these results would translate to more practical settings.

Kurylyak et al. published one of the most highly-cited papers dealing with BP estimation from PPG using a neural network in 2013 [18]. They used a small subset of data from the MIMIC II database to first compute 21 features that describe the shape of an individual PPG cycle in great detail. Correlation between these features and BP was studied and the features were then fed into an artificial neural network (ANN) to train a predictive model. They reported the MAE of 3.80 ± 3.46 for SBP and 2.21 ± 2.09 for DBP. The results are good but only a small undisclosed subset of the MIMIC database was used.

A major recent study was published by Xing et al. in 2016 [19]. They used 69 patients from the MIMIC II database and additional 23 volunteers. Initial strict preprocessing was conducted on all signals to obtain high-quality waveforms. Unlike previous work where temporal features were used, they used the fast Fourier transform (FFT) to extract amplitude and phase features from the waveforms in the frequency domain. These features were again fed into an ANN and good results were reported, in accordance with main standards for BP estimation devices. A special contribution is the proposition of normalization of PPG waveforms across patients, supposedly removing the need for calibration.

We are aware of no work directly using the raw PPG signal as input into deep learning for BP estimation, however, Gotlibovych et al. investigated using raw PPG data for atrial fibrilation detection in 2018 [20] with reasonable success, hinting at the potential of raw signal inputs.

In addition to the reviewed related work, there are a large number of other quality papers published in this domain, however they follow the established approach of using the PPG signal to compute cycle-based features, which are then used to train a regression ML model, so they are not discussed individually.

### 1.4. Our Research in the Context of Related Work

The well established PTT approach is dominant in this field, which is expected, as the underlying mechanisms describing the PTT and BP correlation are formalized and well-tested. The PPG-only approach does offer a lot of advantages and makes sense in the era of wearable devices, mHealth and telemedicine, but is less understood and researched, thus motivating us to focus on only the PPG signal.

A common occurrence in the related work is the usage of either an unavailable privately collected dataset or an unspecified subset of the MIMIC database. On one hand, for privately collected data, this is understandable, since medical data is sensitive and the researchers who collected it may not be able to get permission for sharing. On the other hand, when working with a publicly available dataset such as MIMIC, the selected subset or the procedure to obtain it should be specified. In light of the general crisis of reproducibility in science [21], working with undisclosed or publicly unavailable data can be problematic. This problem was encountered several times by the authors of this paper as well, as both specific data used and the code for the experiments are almost never available from the authors.

It is also common for related work to not compare against a regressor which always predicts the mean of the train data. This is important as it may happen that a selected subset of data has a very small range of ground-truth BP (e.g., a subject with relatively constant BP), in which case the errors will be low, but this does not necessarily signify that the model has learned much more than predicting the mean.

This motivated our research to start from the full publicly available MIMIC database (version III), narrowing it down to a subset of quality waveforms with explicitly described filtering, which got us to our final subset used in training and evaluation. As the database is huge (tens of thousands of subjects, each with potentially several recording sessions), it is also very suitable for use with deep learning, which can use raw signals as input to derive its own features. This is valuable because the related work relies on a rather standard set of features, which require perfect waveforms, heavily relying on the single explicit diastolic peak, which can very often be difficult to detect or even missing. Furthermore, some related work [22] also suggests additional features coming from the first (PPG’) and second derivative (PPG’) of the PPG waveform which are even more difficult to detect consistently, as shown in Figure 1.

We addressed the discussed issues by:

1. Using a large, precisely specified subset of the MIMIC III database with available IDs and the corresponding code for obtaining it, and
2. Directly using the PPG and its derivatives waveforms as input into a novel spectro-temporal residual neural network, which successfully modelled the relationship between PPG and BP. Our proposed neural network architecture is, to our knowledge, the most sophisticated in this field to date, as it takes into account both temporal and frequency information contained in the PPG waveform and its derivatives. The architectural details are described in the later sections and the code for the models is made available.

## 2. Materials and Methods

As mentioned previously, we based our work off of the MIMIC III database [11,23], available at https://physionet.org/physiobank/database/mimic3wdb/. The database contains a myriad of different types of data recorded during hospital admissions of over 30,000 patients aged 16 or above. The median age of adult patients is 65.8 years, 55.9% of patients are male and 44.1% are female. Each patient can have several recordings, which can last from seconds (typically anomalies) to many hours. We were interested in patients for which bedside waveforms were recorded, specifically those that have both PPG and ABP signals. Once the data was cleaned and pre-processed, features were first computed and a classical ML model was trained (Random Forest). Finally, the raw data was fed as input into a deep neural network (DNN) with the aim of predicting SBP and DBP.

### 2.1. Obtaining and Cleaning Raw Data

Since the full database is very large, there were some challenges in initially obtaining the data and subsequently choosing the patients with quality waveforms suitable for machine learning. The upside of such a large quantity of data is the fact that we could afford to be quite strict in our cleaning procedures, since plenty of data remained after every cleaning step.

We initially downloaded the data using a custom DataMiner.sh bash script. In order to download the data in the wanted MATLAB format, the WFDB software package was used [23], specifically the wfdb2mat function. A dedicated server was tasked with downloading the data and saving it to a local storage. We initially noticed that some of the downloaded files were empty or contained an extremely small amount of samples. In the interest of saving space, files under an empirically determined size threshold of 17 kilobytes were deleted by the Cleaner.sh bash script and were not even considered for further cleaning steps. This script also deleted the files which did not contain both PPG and ABP waveforms (specified as *PLETH* and *ABP* in the files). This step brought us down to roughly 10,000 patients.

Afterwards, we conducted a more detailed cleaning procedure, dealing with waveform quality. First, the minimal required length of PPG and ABP was set to 10 min. All the recordings with shorter length were deleted, since we wanted sufficiently long waveforms to contain at least some changes in SBP and DBP. The PPG signal was then normalized to zero mean unit variance and filtered with a 4th order Butterworth band-pass filter, with cutoff frequencies of 0.5 Hz and 8 Hz. Anything below 0.5 Hz can be attributed to baseline wandering, while anything above 8 Hz is high-frequency noise. Afterwards, the signal was again filtered with the aim of removing outliers, using the Hampel filter. This takes a sliding window of seven subsequent PPG samples and computes the median of this window. It then estimates the standard deviation of each sample about the window median. If a sample differs from the window median by more than three standard deviations, it is replaced with the median [24]. The Hampel filter was chosen over median as it is shown to be slightly superior by some related work on signal processing [25] and also because its effects have proven to be satisfactory upon visual inspection. It is important to note that even though some high-frequency noise can be sometimes observed in the ABP waveforms, these were not filtered or pre-processed in any way, since it might slightly affect the signal values, which are needed as the ground truth for SBP and DBP. SBP is obtained as the peak (or the average of detected peaks) in an ABP cycle (or segment) which arises during the systole of the heart, while DBP is the valley between ABP cycles which manifests during the diastole.

The signals were then segmented into cycles, where each cycle corresponds to a single heart beat. The segmentation was conducted using a peak and valley detector proposed by Elgendi et al. [26]. The authors had shown this algorithm to be faster, more efficient and, most importantly, more precise than traditional existing peak detectors used for PPG signal. Upon initial empirical inspection, it worked well on our dataset, but it was very difficult to do a precise evaluation since ground-truth peaks were not known. It could be done by an expert segmenting the PPG cycles by hand via visual inspection, however, such work would be quite demanding.

**Figure 2:**
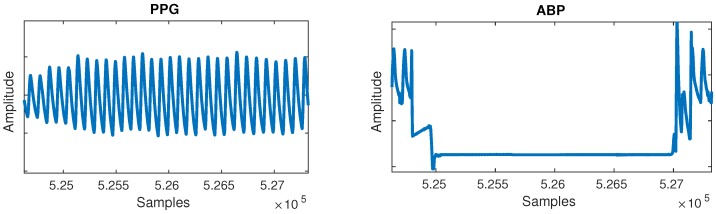
Flat lines anomaly can be observed in the arterial blood pressure (ABP) signal.

**Figure 3:**
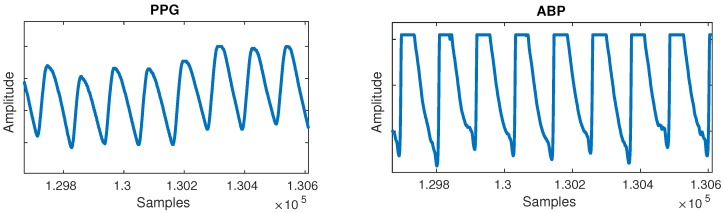
Flat peaks anomaly can be observed in the ABP signal.

The segmentation into cycles was followed by en empirical evaluation of the waveforms. A large number of short random segments of PPG and ABP of random recordings were plotted, which allowed us to identify common post-filtering issues in the waveform morphology. These can mostly be put into two major groups:

1. Flat lines: Flat lines sometimes appeared for long periods of time between normal cycles in both PPG and ABP, as shown in Figure 2. A flat line was detected when three or more consecutive signal samples did not change their value. Such flat lines could be observed in several separate segments of the signal and we postulate they were caused by a periodic sensor anomaly or detachment of sensor. Such areas were useless and were thus cut out from the waveforms.
2. Flat peaks: Similarly, it was common for the ABP waveforms to have flat peaks with top parts missing, as shown in Figure 3. After the PPG was segmented into cycles, peaks were similarly detected by checking if three or more consecutive samples had the same value within a given cycle. The cause was again unknown, but could most likely be attributed to a sensor issue. The peak of the ABP is vital, as its value is the SBP, which is the ground truth needed for machine learning.

As before, we applied a rather strict criterion due to the quantity of the data—if more than 5% of all cycles had flat peaks or if more than 10% of a recording duration consisted of flat lines, we removed this recording from our dataset.

**Figure 4:**
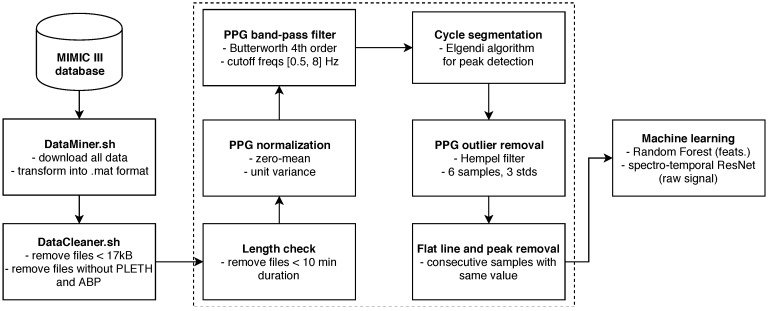
Schematic pipeline of our system.

Finally, any remaining flat lines or flat peak cycles were cut out from the waveforms by simply removing the part of PPG and ABP between the start and end point of remaining flat lines, or, in case of cycles, the full cycle was cut. The entire pipeline is summarized in Figure 4.

**Figure 5:**
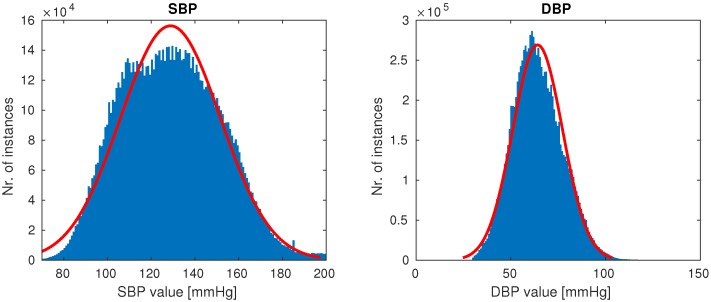
Distributions of systolic blood pressure (SBP) and distolic blood pressure (DBP) in our final data.

Once the cleaning was complete, 510 patients remained, each having at least a single high-quality-waveform recording lasting at least 10 min. Our goal was to create a dataset with the full variation of naturally occuring PPG waveforms (such as those shown in Figure 1), while avoiding anomalies most likely attributed to sensor issues (which were quite prevalent in our dataset). On average, after cleaning a subject had five recordings of length 1.3 h, totalling almost 700 h of training data. The distributions of SBP and DBP of our final data are shown in Figure 5.

The authors of the MIMIC database reported a potential slight delay between the PPG and ABP. Having this in mind, and since the BP does not change substantially under common conditions (excluding arterial bleeding, powerful drugs, etc.), we obtained the ground-truth SBP and DBP by taking the average of detected peaks and valleys in a short segment of ABP—2 s around specific PPG cycle for traditional ML, and 5 s for the PPG segments used in deep learning—which were detected using the same peak detection algorithm as before, since PPG and ABP have quite similar waveforms. A longer segment was taken for deep learning as the spectrogram layer in the neural network required sufficient periodicity in the signal in order to extract meaningful information about the frequency content. The traditional machine learning works on cycles, so a shorter segment was taken (ideally, only the amount corresponding to one PPG cycle would be taken, but it can happen in some edge cases that a systolic peak in ABP is not captured, so a longer length was chosen).

### 2.2. Classical Machine Learning

Since related work has shown that traditional regression models work well with a rather widely used set of features, we conducted such an experiment in order to compare against deep learning. We computed a set of features commonly used across previously discussed related work. Yousef et al. also hinted at the potential usefulness of PPG’ and PPG” [22], so we also attempted to derive features from those. All common features describe the morphology of the PPG waveform on a per-cycle basis. The data was thus initially segmented into cycles and the required points for feature computation (e.g., systolic peak) were determined on the raw PPG cycle and its first derivative. Even though the second derivative was also proposed, its waveforms simply did not exhibit the expected number of peaks, thus making it impossible for those features to be used. All the used features are given in Table 1. Compared to our previous work [27], we semantically expanded our feature set by adding the features from the frequency domain. Most frequency domain features were computed from the PSD obtained using the Welch’s method [28]. At the same time, we removed some features that did not prove valuable in the past.

It is expected in an ideal scenario for a PPG cycle to have two peaks (systolic and diastolic) and PPG’ to have two peaks as well. Our data, however, features dealing with the diastolic notch of the PPG, and the two peaks of PPG’ were difficult to compute due to the diastolic peak being very subtle or even missing, as shown earlier in Figure 1. In these cases, the point where the PPG’ is closest to zero in the area between the systolic peak and cycle end (the point where the PPG signal is the least steep) was taken to compute features dealing with diastolic notch.

The features were finally fed into the default random forest (RF) implementation of scikit-learn using 100 trees, and a leave-one-subject-out (LOSO) experiment was ran, which we describe in more detail in subsequent sections.

### 2.3. Deep Learning

In an attempt to provide a complete representation of our input data to the neural network we used short segments of raw PPG, PPG’ and PPG” as temporal domain inputs into deep learning. Additionally, the frequency-domain information was captured by using spectrograms computed from the temporal input segments. In order to sensibly leverage spectrograms, a segment of a signal containing at least some periodic behaviour is needed. We thus split our signal into 5-s segments, instead of the previously used individual cycles which typically last less than a second. The network was trained to output numerical SBP and DBP, thus solving a regression task.

#### 2.3.1. Neural Network Architecture and Hyperparameters

**Figure 6:**
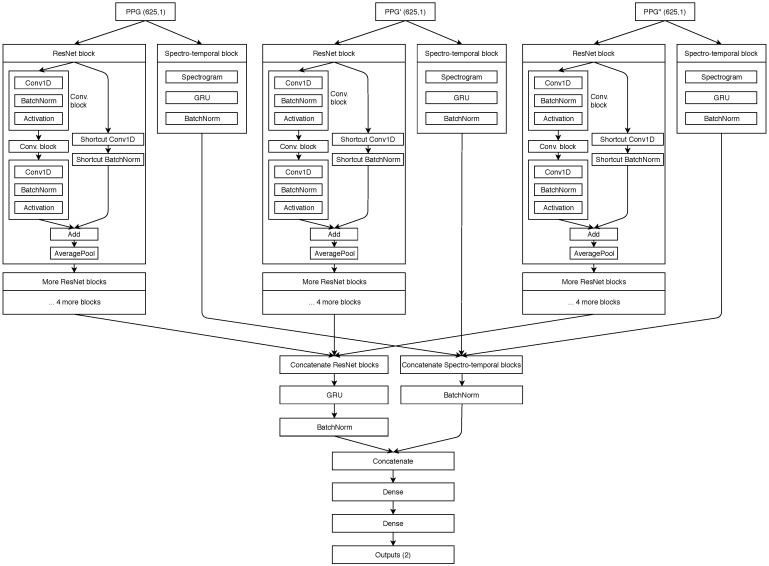
Schematic showing of our neural network architecture.

Our proposed spectro-temporal ResNet is schematically shown in Figure 6. The ResNet idea was originally proposed by He et al. [29] for training very deep networks with the vanishing gradient problem, where the backward error propagation gets too diminished and the first layers do not update their weights sufficiently. Thus, shortcut (residual) connections between larger blocks of layers are proposed. Residual connections were also shown to help in this specific domain of BP estimation from PTT by Su et al. [14].

For each of the three inputs (PPG, PPG’ and PPG”), the temporal representation is extracted by the residual blocks that contain three convolutional (CNN) layers each. Each CNN layer is followed by a batch normalization layer for reducing internal covariate shift, and a ReLU activation layer, which speeds up the training process compared to other activations. Each residual block ends up with an average pooling layer which is used for dimensionality reduction. The network also extracts input-specific spectro-temporal information. The spectral information is extracted by the spectral layer, which computes the spectrogram for each 5-s input segment of the three inputs. The output of the spectral layers is used in gated recurrent units (GRU) to obtain the temporal changes.

The output of each stack of five residual blocks is then concatenated and passed into another GRU layer. Similarly, the output of the spectro-temporal blocks is also concatenated. Both data flows are concatenated for the final time and used in two dense (fully connected) layers. The final output of the network is provided by a ReLu layer, which outputs the predicted SBP and DBP values for the given window. GRU units were used after each spectrogram layer in order to provide additional information about temporal changes of spectrograms for the dense layers later on. Experiments showed this was the best network structure among those tested.

The models were trained with a learning rate of $10^{-4}$ and a decay of $10^{-4}$. The batch size was set to 256 and the maximum number of training epochs was set to 20, with early stopping when no improvement was seen in three epochs. The network parameters including the number of residual blocks, the number of CNN layers per block, the size of the CNN filters, the learning rate and the batch size, were all determined experimentally and can be subject to additional optimization.

The training of the network was fully supervised, by back propagating the gradients through all the layers. The parameters were optimized by minimizing the MAE loss function using the RMSprop optimizer. After the initial training process, the weights of the best epoch were used as the starting point for personalization. In the personalization process, we selected 20% of a given subject’s data to additionally train our network. The rest of the subject’s data was used for the final test. 20% of data has proven to be enough to substantially improve the results, as shown in Table 2.

### 2.4. Experimental Setup

In an attempt to train and evaluate a good general model, a LOSO experiment was conducted. Such an experiment is the most robust in terms of generalization performance, as it is completely person-independent. In each of *n* iterations (n= the number of subjects), the data of n−1 subjects was used for training, while the data of the left out subject was used for testing. MAE was computed in each iteration and the final MAE was given as the average.

An advantage of this experimental setup in contrast to a typical train-test split lies in the fact that the latter can be plagued by overoptimistic or overpessimistic results which might be obtained by taking a very specific split, while in a LOSO setup the results do not depend on the choice of the split.

In the traditional approach we built separate models for SBP and DBP while the neural network was trained to predict both at once, having two outputs.

A disadvantage of this experiment is the extreme computational complexity, especially seen when training the ResNet, as the full network has to be trained *n* times. Considering the amount of data and the complexity of the experiment, a GPU cluster is almost mandatory. In our experiments, we used a combination of a GPU server containing four nVidia 1080Ti 8GB units and a workstation using nVidia Quadro P6000 24 GB units. The described LOSO experiment on the full dataset took over a week.

## 3. Results

The initial person-independent results of the LOSO experiments were subpar compared to related work. Thus, personalization (calibration) was applied, as described at the end of Section 2.3.1, which helped improve the results substantially, as shown in Table 2. The overall errors were computed as the average of individual MAEs in each LOSO iteration. All the results are compared with a dummy regressor, which always outputs the mean of the SBP and DBP from the train set as the prediction.

All the models achieved lower SBP errors compared to the dummy regressor, confirming that some information regarding BP is present in the PPG signal and its derivatives, and that the underlying relationship can be modelled to some extent. However, the dummy regressor displayed low DBP errors, since DBP has lower variance and is generally more stable. Personalization notably improved the results, showing that the relationship between the BP and PPG is subject-dependent.

The comparison between the traditional ML approach using hand-crafted features proposed by related work, and the deep-learning approach using raw signals as input showed better results for the latter. Possible reasons are either that it has more learning capability, that the hand-crafted features do not capture as much information as the DNN can from the raw inputs, or that the commonly used features cannot be successfully used on a largely varied dataset with imperfect waveforms.

Finally, we compared using only PPG as our ResNet input with using PPG alongside its first and second derivative. It can be seen that taking the derivatives decreases the overall MAE by roughly 1 mmHg, hinting at additional useful information contained in the derivative waveforms.

## 4. Discussion and Conclusions

A comparison between our work and the related work discussed in Section 1.2 and Section 1.3 is given in Table 3. In general we find that it is difficult to compare related work in this field due to different evaluation metrics, and different and inadequately specified datasets. Additionally, several authors did not explicitly report whether any data of test subjects was included in the training data (we indicate this by unknown personalization in the table). The lowest errors were achieved on small selected subsets of public or privately collected data with unknown personalization, while work dealing with large scale data (Kachuee et al. [13] and our work) has larger errors, hinting at the difficulty of creating a robust general model on a large dataset. The reason for this may be that the large MIMIC III dataset contains a large variety of subjects as well as potentially different PPG and ABP measurement devices.

Looking at individual related papers, comparing against Chan et al. [12] and Teng et al. [17] was not feasible due to them using ME as a metric. Su et al. [14] used a modern LSTM deep learning architecture, but used the PTT approach compared to using only PPG signal. Kurylyak et al. [18] achieved low errors, but used a quite small amount of unspecified data, namely 15,000 beats were used, which corresponds to just over 3 h of signal (assuming the average cycle length of 0.8 s). They also did not specify explicitly how many subjects this data belonged to, merely the total number of beats, meaning the variation and independence of this data is unknown. Due to extremely small error reported by Xing et al. [19], we attempted to reproduce their work. We could not do so completely, and our errors using a similar network architecture and inputs were larger. The fact that our DNN achieved results comparable to those by Kachuee et al. [13], despite requiring only a single PPG signal compared to using the PTT approach, suggests that our network architecture is well suited to this problem. As far as we know, no work conducted so far has used the raw PPG and its two derivatives as input into a neural network to successfully estimate BP. This is important, as it shows on a large dataset that BP evaluation is feasible using only the PPG signal. Additionally, the proposed neural network architecture allows for fusion of three inputs, as well as frequency and temporal information of each input.

In addition to the novel DNN architecture, another contribution is the fact that the procedure for filtering the data to obtain our subset is described in detail and the final subjects used in training and evaluation are available. The DataMiner.sh, Cleaner.sh and MATLAB pre-processing code are also made available by the authors at https://github.com/gasper321/bp-estimation-mimic3 alongside the Python code of our neural network model definition, which allow researchers to further build upon our work and enables them comparison with one another. Note that this code is experimental and used for research, not robust and production-ready.

The main limitation lies in the personalization requirement, meaning that some data with ground truth is required for the model to be adapted to each user. In practice this would mean the user has to measure their PPG and SBP/DBP with a high-quality cuff-based device and then give this data to the model for personalization. While this requires some effort, it seems a reasonable cost for being able to monitor BP without any user action afterwards. The computational requirement of personalization is a couple of minutes on a GPU, which is not a problem on a server, but currently probably not feasible on a monitoring device itself. As an additional limitation, very noisy data may be problematic, especially regarding the derivative waveforms, as their shape gets extremely malformed.

The standard for the validation of blood pressure measuring devices proposed by the Association for the Advancement of Medical Instrumentation, European Society of Hypertension and International Organization for Standardization [ref] requires that the estimated probability of a tolerable error (≤10 mmHg) is at least 85%. Our results without personalization certainly do not meet this requirement, but the results with personalization come close (the average error is tolerable, although this is true for less than 85% of individual errors). With some modest improvements, personalized BP estimation with a single PPG sensor could thus be accurate enough for informative home use.

PPG-based BP estimation offers greater comfort and—in the home setting—potentially improved user adherence to this important measurement. While the MIMIC III database does not provide information on the devices with which the PPG signal was obtained, they are probably fingertip blood oxygen saturation monitors. They are certainly suitable for one-shot measurements, and patients are sometimes monitored with such devices continuously, so BP could be monitored at the same time. Fingertip devices are generally not suitable for ambulatory use, so different devices would have to be used for unobtrusive continuous BP monitoring. Wristbands, which are the most widespread device equipped with PPG sensors, currently provide inferior PPG signal [27], although other sensors—such as cameras [30]—are being explored for this prupose as well. In summary, we believe the results presented in this paper suggest that BP estimation with a single PPG sensor may be suitable for one-shot BP measurements, while their applicability to continuous BP monitoring is limited.

## Figures and Tables

**Table 1:** Features calculated from PPG on a per-cycle basis.

Domain	Features
Temporal	$T_{c}$. Cycle duration time $T_{s}$. Time from cycle start to systolic peak $T_{d}$. Time from systolic peak to cycle end $T_{steepest}$. Time from cycle start to first peak in PPG’ (steepest point) $T_{diaNotch}$. Time from cycle start to second peak in PPG’ (dicrotic notch) $T_{SysToDiaNotch}$. Time from systolic peak to dicrotic notch $T_{diaToEnd}$. Time from dicrotic notch to cycle end Ratio. Ratio between systolic and diastolic amplitude
Frequency	*Three largest magnitudes.* Three peaks with the largest magnitude from the PSD were considered. These tell us the dominant frequencies in the cycle. Both the magnitude values and the frequencies (in Hz) were taken as features. *Energy.* Calculated as the sum of the squared fast Fourier transform (FFT) component magnitudes. The energy was then normalized by dividing it with the cycle length. (1) $energy=\frac{1}{N}\sum_{n=0}^{N-1}{\left|x\left(n\right)\right|}^{2},$ where x(n) is the *n*-th FFT component and *N* is the parameter specifying the number of FFT components to compute. *Entropy.* Calculated as the information entropy of the normalized FFT component magnitudes. (2) $entropy=-\sum_{n=0}^{N-1}x\left(n\right)\log\left(x\left(n\right)\right)$ *Binned distribution.* A normalized histogram, which is essentially the distribution of the FFT magnitudes into 10 equal sized bins ranging from 0 Hz to 62.5 Hz. *Skewness and kurtosis.* These describe the shape of the cycle. More precisely, skewness tells us about the symmetry while kurtosis tells us about the flatness.

**Table 2:** Mean absolute errors (MAEs) achieved by classical ML and deep learning with and without personalization.

**Leave-One-Subject-Out (LOSO) Experiment (5-s Windows of Raw Signal as Instances)**		
	**MAE for SBP [mmHg]**	**MAE for DBP [mmHg]**
Dummy (mean of training)	19.66	10.64
ResNet (raw PPG, no personalization)	16.39	13.41
ResNet (raw PPG, with personalization)	10.52	7.67
ResNet (raw PPG + PPG’ + PPG”, no personalization)	15.41	12.38
**ResNet (raw PPG + PPG’ + PPG”, with personalization)**	**9.43**	**6.88**
**LOSO Experiment (Per-Cycle PPG Features as Instances)**		
	**MAE for SBP [mmHg]**	**MAE for DBP [mmHg]**
Dummy (mean of training)	19.17	10.22
Random Forest (features, no personalization)	18.34	13.86
Random Forest (features, with personalization)	13.62	11.73

**Table 3:** Comparison with well-established related work in terms of data used, methodology and errors.

Author	Data Used	Method Used	Personalization	Error
Chan et al. [12]	Unspecified proprietary data	PTT approach, classical ML (linear regression)	Yes	ME of 7.5 for SBP and 4.1 for DBP
Su et al. [14]	Proprietary data (84 subjects, 10 min each)	PTT approach, deep learning (long short-term memory (LSTM))	Unknown	RMSE of 3.73 for SBP and 2.43 for DBP
Kachuee et al. [13]	MIMIC II (1000 subjects)	PTT approach, classical ML (AdaBoost)	Optional	MAE of 11.17 for SBP and 5.35 for DBP
Teng et al. [17]	Proprietary data (15 subjects, 18 seconds each)	Temporal PPG features, classical ML (linear regression)	Unknown	ME of 0.21 for SBP and 0.02 for DBP
Kurylyak et al. [18]	MIMIC (15,000 beats)	Temporal PPG features, deep learning (fully-connected artificial neural network (ANN))	Unknown	MAE of 3.80 for SBP and 2.21 for DBP
Xing et al. [19]	MIMIC II (69 subjects) and proprietary data (23 subjects)	Frequency PPG features, deep learning (fully-connected ANN)	Unknown	RMSE of 0.06 for SBP and 0.01 for DBP
Our work	MIMIC III (510 subjects)	Temporal and frequency features of PPG, PPG’ and PPG”, deep learning (spectro-temporal ResNet)	Yes	MAE of 9.43 for SBP and 6.88 for DBP

## Abbreviations

The following abbreviations are used in this manuscript:

ABP	arterial blood pressure
ANN	artificial neural network
BP	blood pressure
CV	cross validation
CVDs	cardiovascular diseases
DBP	diastolic blood pressure
ECG	electrocardiogram
FFT	fast fourier transform
GDPR	general data protection regulation
HR	heart rate
LED	light-emmiting diode
LOSO	leave one subject out
LSTM	long short-term memory
MAE	mean absolute error
ME	mean error
PPG	photoplethysmogram
PPG’	1st derivative of photoplethysmogram
PPG”	2nd derivative of photoplethysmogram
PPT	pulse transit time
PSD	power spectral densitiy
PWV	pulse wawe velocity
RNN	recurrent neural networks
SBP	systolic blood pressure
WHO	World Health Organisation
